# Cerebellar Stroke Occupational Therapy and Physical Therapy Management from Intensive Care Unit to Outpatient: A Case Report

**DOI:** 10.7759/cureus.1949

**Published:** 2017-12-14

**Authors:** Christopher M Wilson, Christina L Mitchell, Katherine M Hebert

**Affiliations:** 1 Physical Therapy Program, School of Health Sciences, Oakland University; 2 Rehabilitation Services, Beaumont Hospital, Troy, Mi; 3 Physical Therapy Program, School of Health Sciences, Oakland University, Rochester, Mi

**Keywords:** cerebellar stroke, multimodal sensory stimulation, early mobility, cerebellar diplopia, stroke, cerebellum, palliative, icu, physical therapy, occupational therapy

## Abstract

Cerebellar stroke increases the risk of extensive physical disability and long-term institutionalization. The purpose of this case report is to describe the 14-month longitudinal rehabilitation management and outcomes from the intensive care unit, inpatient rehabilitation unit and outpatient care of a patient after cerebellar stroke. A goal of this case report is to provide rehabilitation clinicians with a long-term perspective and understanding of the course of recovery for a patient after cerebellar cerebrovascular accident or related injury. A 51-year-old healthy athletic female experienced acute bilateral cerebellar infarcts with subsequent craniotomy to remove infarcted areas. The patient had postoperative hemorrhages and hydrocephalus and was deemed to have a poor prognosis. Multimodal sensory stimulation and early mobility was performed until conventional neuromuscular reeducation interventions could be tolerated. Primary deficits included decreased proximal strength, whole body ataxia, vertical diplopia, dysphagia, difficulty communicating, and emotional lability. Fourteen months after the initial infarcts, the patient was able to reside in her own home with her husband, ambulate, and stand with assistance and perform most activities of daily living with standby or set-up assistance. This patient made significant progress toward safety and mobility and was able to return home despite the early discussion about a poor prognosis and a palliative care consultation. The complex, intensive course of rehabilitation elicited slow, steady, consistent gains. The patient’s motivation and family involvement likely facilitated optimum outcomes.

## Introduction

Cerebellar infarction represents approximately 2.3% of acute strokes overall [1]. Larger cerebellar infarcts produce symptoms and signs localizing to the brainstem, such as diplopia, dysarthria, limb ataxia, dysphagia, and weakness or numbness. Approximately 10% of patients with cerebellar infarction can present with isolated vertigo, that is, vertigo with no localized or diagnostic findings on motor, sensory, reflex, cranial nerve, or limb coordination upon examination. Most (96%) cerebellar strokes are infarcts of the medial branch of the posterior inferior cerebellar artery (PICA) [2]. Mortality after cerebellar stroke is higher than that of other vascular territories. This is generally due to concomitant brainstem infarction or compressive hydrocephalus, rather than cerebellar infarction in itself [3].

Kelly et al. [4] examined functional recovery after cerebellar stroke and found that outcomes were positively correlated with admission functional status into inpatient rehabilitation. Functional gains were negatively correlated with presence of pre-existing comorbidities, an altered level of conciousness upon intial presentation, and the infarct occuring at the superior cerebellar artery. Mean admission functional independence measure (FIM) score was significanlty lower for hemmhorages as compared to infarctions (p=0.006) [4]. Kelly et al. reported a mean improvement in FIM score from admission to discharge of 70 to 93 for cerebellar infarcts [4]. Cerebellar hemmhorage FIM scores also improved from 43 to 74. There is a gap in the literature as it relates to long term progress and comprehensive rehabilitation management for patients after cerebellar stroke.

The purpose of this case report was to describe the longitudinal management for occupational therapy (OT) and physical therapy (PT) for a patient with a cerebellar stroke over a 14-month treatment course. This case report will provide a long term perspective and outcomes after a complex and lengthy rehabilitation course to guide rehabilitation clinicians in long-term and short-term management after a cerebellar stroke. Although speech language pathology (SLP), nursing, social work, medical care, and recreation therapy were integral to the recovery of this patient, the focus of this article was on OT and PT services. According to the Beaumont Health Institutional Review Board (Royal Oak, MI, USA), a case report does not constitute human subjects research. The patient and her spouse provided written informed consent to publish this case report and the associated photographs.

## Case presentation

A 51-year-old healthy female arrived at the emergency center presenting with acute dizziness, a severe headache, diaphoresis, diffuse photophobia, nausea, and emesis. The patient reported that she had been doing pushups during a televised total body workout and was suddenly unable to lift herself up. A head computerized tomography (CT) demonstrated that there was no acute intracranial event. Her symptoms were initially believed to be a vasovagal reaction. She was bradycardic at 25 to 39 beats per minute. The emergency room plan was to monitor orthostatic vital signs and aggressively hydrate overnight. The next day a repeat head CT demonstrated acute ischemia of the left cerebellum mainly in the PICA distribution. Upon this finding, the patient was admitted into the hospital diagnosed with an acute cerebrovascular accident (CVA) in the cerebellum.

Prior to the cerebellar CVA, the patient was active and independent with both basic and advanced activities of daily living (ADL). Her social history included being a former smoker for eight years, consuming two to three drinks per week, and completing high intensity exercise six times a week (mostly aerobic). Her health history included a patent foramen ovale, two superficial venous thrombi, and a history of depression. Prior to admission, her medications included aspirin 81 mg daily, estradiol 0.5 mg, ibuprofen 200 mg, a daily multivitamin, nystatin suspension, and 500 mg of vitamin C. The patient was living with her spouse and four children in a two story home with a two-step entry. Her bedroom and full bath were on the second floor with a half bath on the first floor. Two of her children were attending college and two teenaged children were living at home. Her husband worked full time as an engineer but had a very flexible schedule and she had a large social network for support after discharge.

Initial emergency room visit and symptom progression

Based on her diagnosis and symptoms, the patient received an evaluation by SLP as well as PT and OT. She was found to have double vision, dizziness when sitting up, and complained of a headache that she subjectively rated as a 7/10 (0 = no pain and 10 = worst pain). She demonstrated an initial score of three on the National Institute of Health Stroke Scale and was placed on the hospital stroke protocol. She had normal range of motion (ROM), muscle strength, tone and sensation. It was found that her coordination was impaired, her gait was ataxic without an assistive device and supervision was required for bed mobility and transfers. The patient scored a 2/6 (0=no impairment, 6=severe disability/bedridden) on the modified Rankin scale. A PT treatment plan was established for balance retraining during her initial visits but subsequent medical events did not allow for PT interventions to be performed.

**Figure 1:**
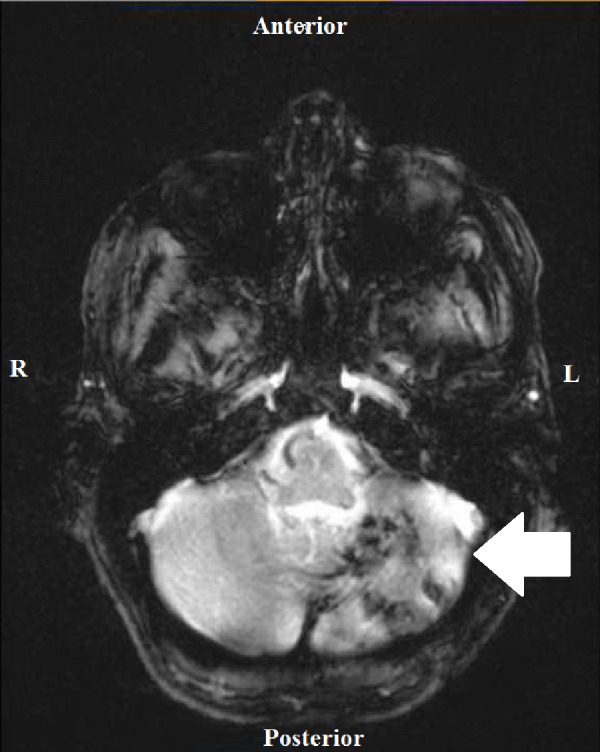
Brain Magnetic Resonance Imaging after Craniotomy Brain MRI (T2 Weighted) without gadolinium completed two months after left occipital craniotomy and evacuation of infarcted left cerebellar tissue. Images demonstrate left cerebellar subacute and chronic infarctions as well as an area of acute infarction within the pons.

Four days after her admission, she was found face down in bathroom after ambulating there independently. She was alert, oriented, and responding appropriately. A head CT revealed hydrocephalus with dilation of the lateral ventricles including temporal horns and third ventricle, along with fourth ventricle compression and brain stem compression due to edema of a left cerebellar infarct. She became progressively more lethargic and almost non-verbal, but still was able to follow commands. The patient was then transferred to the intensive care unit (ICU) and had emergency surgery for a left suboccipital craniotomy with removal of the infarcted areas in the cerebellum. After surgery, a head CT demonstrated a new hemorrhage and gas within the operative cavity along with an increased mass effect on the left side of the fourth ventricle which was completely effaced with moderate obstructive hydrocephalus. Later in the day the patient demonstrated a reduced level of consciousness and was taken to the operating room again where a right frontal ventriculostomy was performed to decrease the hydrocephalus. Postoperative cerebellar damage is depicted via magnetic resonance imaging (MRI) on Figure 1. She subsequently had a posterior fossa decompression and she was deemed to have an overall poor prognosis. 

Three days after her fall and subsequent surgeries, the patient remained orally intubated and unresponsive. The patient’s intracranial pressure (ICP) was found to be elevated and she had an external ventricular drain (EVD) placed for therapeutic drainage of her cerebrospinal fluid (CSF). She subsequently developed a hemorrhage surrounding her EVD in the right frontal lobe and a hemorrhage within the right lateral ventricle. The next day she underwent surgery to reopen her suboccipital craniotomy for evacuation of a clot. Seven days after her fall and surgery, she remained intubated and on a ventilator. By this time, her medical status became more stable overall, however she remained verbally unresponsive and demonstrated a positive Babinski sign on the left foot. In light of her poor prognosis, the patient received a palliative care consultation to assist in coordinating care and decision making 11 days after her fall and surgery. Twelve days after her initial fall, a tracheostomy was completed and she remained on ventilator support. Two days after the tracheostomy, she was successfully transferred to supplemental oxygen at 28% FiO_2_ via a tracheostomy collar.

Results and interventions during occupational therapy and physical therapy

**Figure 2:**
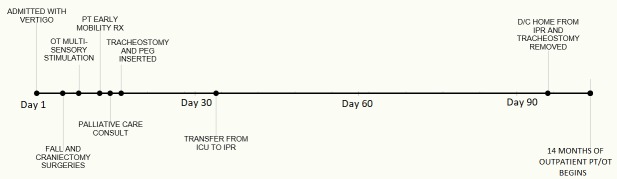
Intensive Care Unit and Inpatient Rehabilitation Unit Timeline OT = occupational therapy, PT = physical therapy, RX = treatment, PEG = percutaneous endoscopic gastrostomy, ICU = intensive care unit, IPR = inpatient rehabilitation unit, D/C = discharge

**Figure 3:**
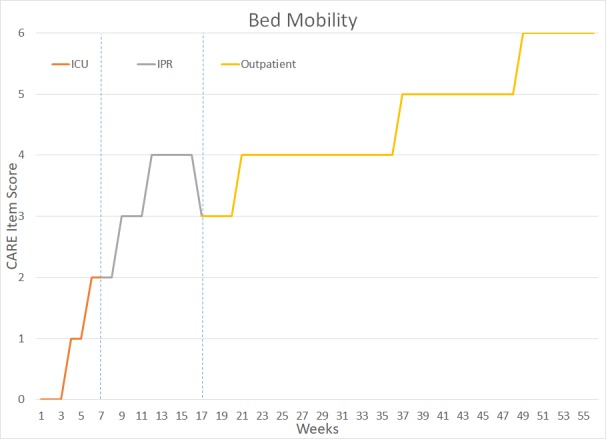
Bed Mobility Performance Week 1-6 = Intensive Care Unit (ICU) Week 7-16 = Inpatient Rehab Unit (IPR) Week 17-56 = Outpatient Occupational Therapy and Physical Therapy

**Figure 4:**
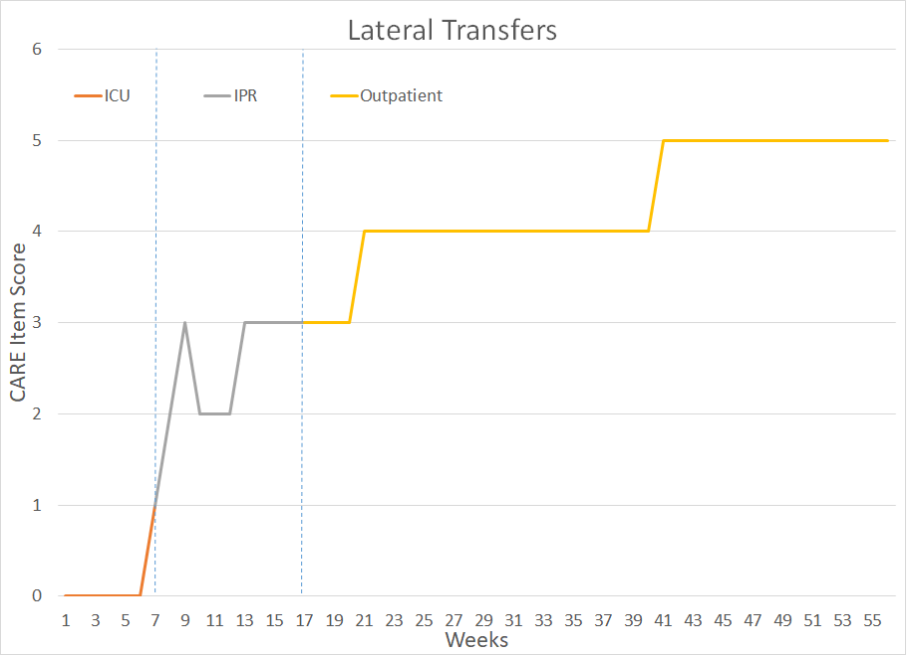
Lateral Transfers Performance Week 1-6 = Intensive Care Unit (ICU) Week 7-16 = Inpatient Rehab Unit (IPR) Week 17-56 = Outpatient Occupational Therapy and Physical Therapy

**Figure 5:**
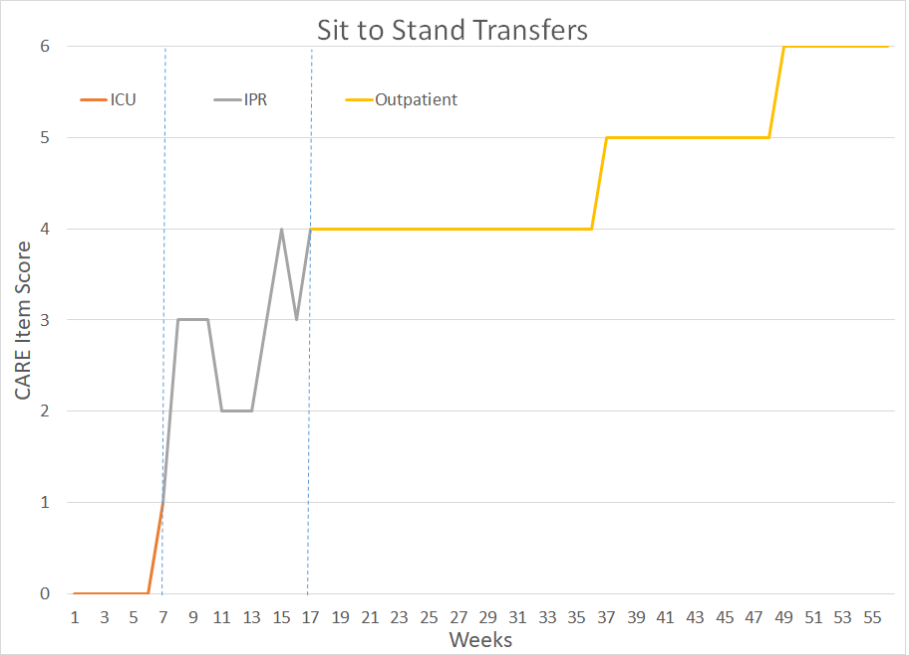
Sit-To Stand Transfers Performance Week 1-6 = Intensive Care Unit (ICU) Week 7-16 = Inpatient Rehab Unit (IPR) Week 17-56 = Outpatient Occupational Therapy and Physical Therapy

**Figure 6:**
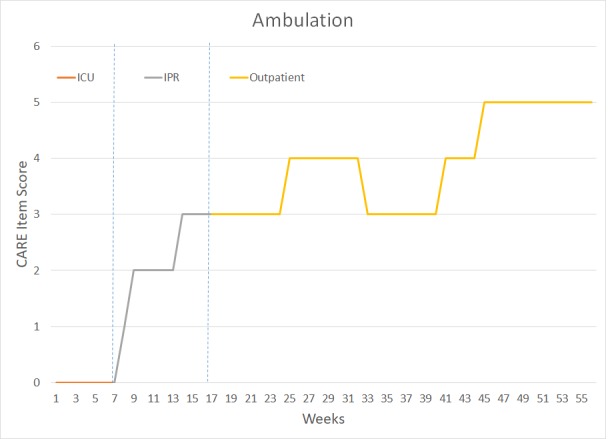
Ambulation Performance Week 1-6 = Intensive Care Unit (ICU) Week 7-16 = Inpatient Rehab Unit (IPR) Week 17-56 = Outpatient Occupational Therapy and Physical Therapy

**Figure 7:**
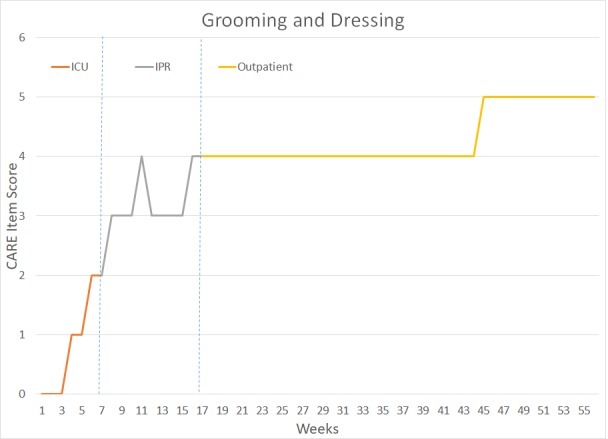
Grooming and Dressing Performance Week 1-6 = Intensive Care Unit (ICU) Week 7-16 = Inpatient Rehab Unit (IPR) Week 17-56 = Outpatient Occupational Therapy and Physical Therapy

The patient underwent a total of 14 months of PT and OT – six weeks in the intensive Care Unit (ICU), approximately 10 weeks in Inpatient Rehabilitation (IPR), and 41 weeks of outpatient rehabilitation. Figure 2 outlines key milestones within her ICU and IPR rehabilitation. Figures 3-7 depict the patient’s overall progress using the Continuity Assessment Record and Evaluation (CARE) item scoring for major activities of daily living (ADL) and physical mobility tasks across the continuum of care. See Table 1 for CARE item scoring and interpretation.

**Table 1:** Continuity Assessment Record and Evaluation (CARE) Item Coding Key https://www.cms.gov/Medicare/Quality-Initiatives-Patient-Assessment-Instruments/Post-Acute-Care-Quality-Initiatives/Downloads/CARE-Institutional-Admission-Assessment-Tool.pdf

Score	Description
6	Independent – Patient completes the activity by him/herself with no assistance from a helper.
5	Setup or clean-up assistance – Helper SETS UP or CLEANS UP; patient completes the activity. Helper assists only prior to or following the activity.
4	Supervision or touching assistance – Helper provides VERBAL CUES or TOUCHING/STEADYING assistance as patient completes activity. Assistance may be provided throughout the activity or intermittently.
3	Partial/moderate assistance – Helper does LESS THAN HALF the effort. Helper lifts, holds or supports trunk or limbs, but provides less than half the effort.
2	Substantial/maximal assistance – Helper does MORE THAN HALF the effort. Helper lifts or holds trunk or limbs and provides more than half the effort.
1	Dependent – Helper does all of the effort. Patient does none of the effort to compete the task.
0	Activity not attempted/completed due to medical condition, safety concerns, environmental constraints, or patient refused.

Intensive Care Unit Rehabilitation

The patient was in the ICU for a total of six weeks and received 19 visits of OT, 12 visits of PT, and 17 visits of SLP. The OT sessions lasted 30 to 45 minutes while PT sessions lasted 30 minutes. Occupational therapy was focused on multimodal sensory stimulation (MSS) and once the patient was responsive enough to benefit from early mobility, PT was initiated. Occupational therapy was performed immediately prior to PT to optimize wakefulness and participation in early mobility activities. After PT/OT treatment plans were established, they prescribed complimentary restorative aide services (trained technicians supervised by OTs and PTs who assist patients with exercises, general mobility and ROM) for a total of eight times while in the ICU.

Occupational therapy evaluation, interventions and outcomes: Three days after the initial fall and surgery, the patient was evaluated by OT. Initially she presented as minimally responsive with her eyes closed throughout the session; she was non-verbal with no attempts to communicate and was flaccid in both arms and legs with mild edema, and dependent with all ADL and functional mobility. Goals of OT were to increase environmental engagement and response to stimuli to begin early mobility with physical therapy.

The majority of the ICU OT treatment sessions consisted of MSS, which is an application of a series of sensory techniques that are customized in occurrence and amount in order to increase arousal and awareness to elicit a purposeful behavior. MSS is also known as multi-sensory stimulation or “coma stim” [5]. The MSS therapy consisted of interventions such as wiping a cold washcloth along the patient’s face, neck and wrist and clapping loudly near the patient’s ears. The patient initially responded to the MSS with an elevated heart rate and blood pressure, and then began communicating by nodding and biting on her endotracheal tube, turning her head away, and wiggling her fingers. Subsequent OT treatments in the ICU included bed mobility and ADL retraining for which the patient was initially dependent. As the patient became more responsive, neuromuscular re-education techniques were used such as the therapist placing the patient’s right hand on her hair and asking her to lower her arm to touch her eyes, nose and mouth, use of a universal cuff on the right upper extremity to assist with brushing her teeth and preparing to eat. Other various treatments that were utilized in the ICU included grasping and releasing cones and sponges, muscle tapping/brushing, a bilateral pectoral myofascial release, joint compression in order to increase proprioceptive output, visual tracking, and the application of L’nard boots (AKA preventative ankle foot orthoses) to prevent foot drop and a right eye patch to address double vision. In the last week of the patient’s stay in the ICU, she demonstrated moderate to maximal assistance with grooming, she was dependent with dressing and toileting, required maximal assistance with side-to-side bed mobility and supine-to-sit transfers, and continued to have poor static and dynamic seated balance. At discharge from the ICU, her alertness improved and she displayed consistent command following and was progressing to using one or two fingers and head motions for no/yes with good accuracy and she was consistently alert and oriented to one to two.

Physical therapy evaluation, interventions and outcomes: Physical therapy treatments were initiated 30 days after the patient’s initial fall and eight days after OT was initiated in the ICU. Key evaluation findings demonstrated full passive range of motion but lack of purposeful movement, however the patient did occasionally move her arms and legs spontaneously against gravity. Initial PT treatments consisted of passive range of motion (PROM) and progressing to active assistive range of motion (AAROM) and stretching. The physical therapist also provided a home exercise program and fall prevention education to the family and nursing. In addition, early mobility procedures were initiated including progressive sitting up in bed, at the edge of the bed and tilt table [6]. At week five, the tilt table was introduced with intervals of two to ten minute holds to allow her vitals and symptoms to stabilize. At the end of her ICU stay (or after a week-and-a-half of treatment) she was able to tolerate a 70 degree tilt-table angle for 45 to 55 minutes three times per day.

Inpatient Rehabilitation Unit

The patient was directly admitted to the IPR unit after a six week ICU stay. During inpatient rehabilitation, the patient received a total of 54 visits of OT, 51 visits of PT, 47 visits of SLP and 22 visits of recreation therapy. The patient was in the inpatient rehab setting for 10 weeks and consistently maintained the admission criteria for three hours per day averaging five to seven days per week. Throughout the IPR stay, the patient’s husband was active, engaged and participated in the entirety of the educational process of rehabilitation.

Occupational therapy evaluation, interventions and outcomes: The OT evaluation found decreased muscle strength grossly to both shoulders at 1/5, elbow and wrist strength was 2/5, while the forearm and hands were 3-/5. The patient demonstrated hypotonicity which was accentuated proximally, as well as dymetria and dysdiadochokinesia of both arms. She demonstrated truncal ataxia resulting in poor sitting balance and required total assist with upper body and lower body dressing, bathing, toileting, and all bed mobility. The patient required total assist of three clinicians during a partial-stand bed-to-chair transfer using a sliding board. She was unable to tolerate static sitting without total assistance of two people to maintain her balance. She had not advanced to wheelchair propulsion by this time and was still utilizing a trach collar with humidified air. She required a soft cervical collar during transfers and upright activities as she did not have the neck extensor strength to lift her chin off of her sternum against gravity. The patient denied any pain during the IPR series of visits with the exception of pain reproduced during weight bearing on the upper extremities during sitting at the edge of the mat.

Her initial series of OT visits emphasized control of upright sitting with cueing as well as utilizing kinesiology tape to facilitate cervical extension. After two weeks in IPR, the patient no longer required the soft collar during transfers. The patient participated in upper extremity ROM progressing from gravity-eliminated/AAROM and also received scapular mobilizations. Vision treatments included application of tactile and auditory stimulation for coordination of eye active range of motion (AROM) and a trial of clear eyeglasses with a unilateral gauze patch over the right eye for diplopia. The patient also performed gaze stabilization exercises as well as progressive transfer activities incorporating tracking and planning. By week four in IPR, the patient demonstrated improvement in bed mobility from maximum to moderate assist with improved motor control. She had also improved to requiring moderate assistance during static sitting balance. At this time, the patient was able to perform a standing pivot transfer from a bed to a chair with maximum assistance of one person.

The patient demonstrated static sitting balance of 40 seconds with steadying assist. By week six, the patient was able to propel the wheelchair 30 feet utilizing only her legs for propulsion as she was unable to grasp or elevate her upper extremities to the propulsion level of the wheelchair at this point; this improved to 50 feet by week seven of IPR. During week seven and eight in IPR, specific treatments included emphasizing wheelchair seating and positioning with tasks such as leaning forward in her wheelchair to rest her elbows on tops of her thighs and returning to upright as well as wheelchair push-ups and lower body dressing. Vision treatments included activities such as visual fixation on targets with lateral head movements, letter and number reading, and utilization of the Nintendo Wii emphasizing hand-eye coordination.

By week eight, the patient only required moderate assistance with transfers and brief static standing. Her wheelchair sitting tolerance improved to 20 minutes. By week nine, OT treatments emphasized reaching forward and down to the ground while in a wheelchair with return and upper extremity stretching and strengthening focusing on optimizing functional reaching activities. Intervention strategies also included reaching activities while in a standing position utilizing steadying assist and incorporating hand-eye coordination. During her last week in inpatient rehabilitation, she displayed active left shoulder elevation of 110° and 140° on the right and gross left arm strength of 3/5 while the right arm was grossly 3+/5. The patient was rolling side-to-side with standby assist and her static sitting balance was also standby assist. Static standing required minimum assist while dynamic balance required maximal assistance. Her tub/shower and bed-to-chair transfers required moderate assistance.

Physical therapy evaluation, interventions and outcomes: The patient’s lower extremity muscle strength via manual muscle testing on initial admission into IPR was 2-/5 for hip flexors while knee extensors, knee flexors and dorsiflexors were 3/5. As noted in the OT evaluation, the patient required maximum assistance/total assistance of two to three people and utilized a cervical collar to prevent neck flexion during transfers. The patient demonstrated a standing tolerance of five to ten minutes with maximum assistance. During exercises the patient required continuous verbal cueing and emphasis on breathing techniques. The initial series of lower extremity exercises were AAROM and as the patient progressed to tolerating increased weightbearing and upright activity, open chain activities were modified to be performed in closed chain in varying planes of movement. Upon week two, the patient received assistance with standing activities while using a standing frame and SARA Plus mechanical standing unit (ArjoHuntleigh, Addison, IL http://www.arjohuntleigh.us/) for assisted pre-gait activity with moderate assistance.

**Figure 8:**
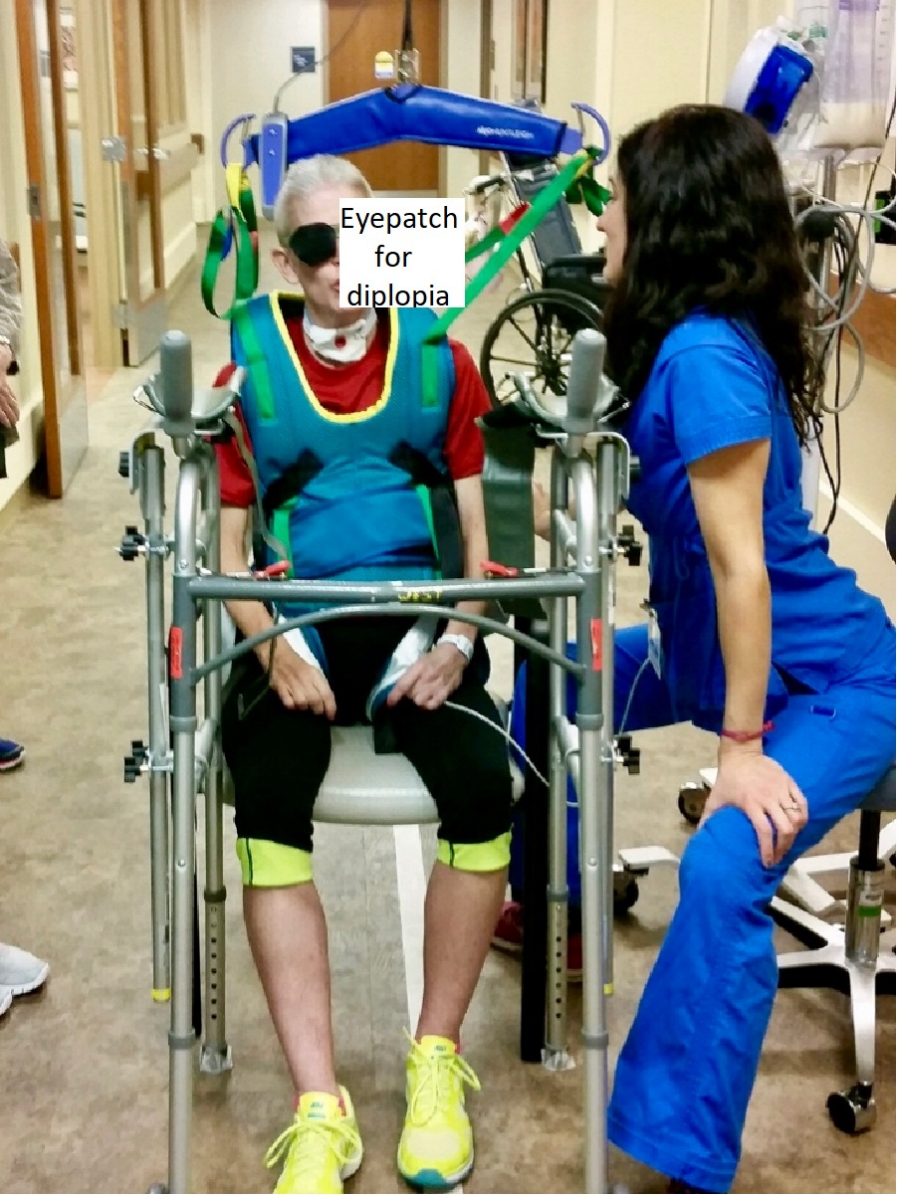
Rest Break during Gait Training Seated rest break during gait training in inpatient rehabilitation unit.  Gait training was performed using a rolling walker and ceiling-mounted harness for partial unweighting and fall prevention

By week two, the patient was tolerating light ankle weights of one pound for long arc quads and progressing to active range of motion from active-assistive. Throughout the treatment sequence, the patient was assisted in use of a recumbent elliptical machine; this initially focused on the lower extremities but as the patient’s grasp improved, upper extremities were also incorporated. The patient also transitioned to using a mechanical ceiling-mounted gait harness and was able to tolerate ambulation over 18 m (60 ft) with maximum assistance of two people. The patient was propelling her wheelchair with moderate assistance for 30 m (100 ft) forward and backwards (Figure 8). During this propulsion, she demonstrated limitations with trunk and hand control that affected her ability to maintain a midline position. 

By week four of PT in IPR, the patient had progressed to gait training activities utilizing the LiteGait assisted gait system (LiteGait, Tempe AZ, http://www.litegait.com/) and the patient was able to support 60-90% of her body weight with 4.5-8 kg (10-40 lbs) of assistance from the harness for 92 m (301 ft) on even surfaces. This progressed to 227 m (745 ft) later that week utilizing a platform rolling walker. By week four, she was able to participate in sit-to-stand transfers with minimum to moderate assistance however pivot transfers and sustained standing continued to require maximum assistance. By week six, the patient was performing gait training with the SARA Plus walking sling for distances up to 366 m (1,200 ft) with three standing rest breaks with moderate assist with 0-11 kg (0-25 lbs) of unweighting at 0.22 m/s (0.5 mph). She began unsupported gait training with a platform walker and bilateral hand splints but required two seated rest breaks over 60 m (190 ft).

Upon week seven, the patient was able to sit at the edge of the mat with her hands on her lap for 90 seconds with supervision while external perturbations and isometric resistance were applied. The patient was also able to begin car transfer training with moderate to maximum assistance. She was now able to propel her wheelchair 24 m (80 ft) but as noted above in the OT section, this task mainly utilized her lower extremities due to poor handgrip and upper extremity strength. The patient performed sit-to-stand transfers with minimum assistance and required moderate to maximum assistance for overall gait training. Standing pivot transfers had progressed to minimum assistance and she was able to roll in bed with steadying assist, however, she still required moderate assistance for other bed mobility tasks. The LiteGait for gait training continued to be emphasized and the patient was able to ambulate 426 m (1400 ft) with 0-11 kg (0-25 lbs) of unweighting at 0.22-0.27 m/s (0.5-0.6 mph). During gait training with her platform walker, she no longer required a hand splint for the right upper extremity but still required this for the left hand to facilitate grasp. She was able to begin stair training with a 15 cm (6 in) step with maximum assistance of two people.

By week nine, the patient was safe enough to perform gait training with one person assistance without a wheelchair following her. She was able to manage her rolling walker independently and was displaying improvement in balance during dynamic standing activities and stair training. The patient’s therapeutic exercise had subsequently progressed to standing heel and toe raises, marching, mini-squats, ankle pumps, seated alternating hip flexion, supine bridging and balance and core strengthening activities while reaching outside of her base of support. The patient’s sit-to-stand transfers had improved to minimum/moderate assist (depending on fatigue) and her bed mobility also required only minimum assistance. Upon discharge from IPR, the patient was sidestepping in the parallel bars 9 m (30 ft) for two repetitions with moderate assistance. The patient was able to perform wheelchair propulsion using both legs and arms for 46 m (150 ft) with standby assistance. She was able to ascend and descend a 15 cm (6 in) step four times using a handrail with maximum assistance of two individuals. She was able to perform a five-times-sit-to-stand test in 33 seconds with minimum assistance in the parallel bars. By week 10, the patient could perform daily bathing, grooming, showering, and functional mobility tasks with moderate assistance from her husband without cues from the therapists. This allowed for safe discharge to her adapted home environment. Home adaptations included a wheelchair ramp and was discharged to home with a wheelchair, platform walker, shower chair, tub transfer bench and subsequently received outpatient PT and OT after a one week gap in treatment due to the holidays.

Outpatient Rehabilitation

Upon initiation of outpatient PT and OT seventeen weeks after her fall and craniotomy surgery, the patient presented with decreased core strength requiring a specialized wheelchair for positioning and stability. During outpatient rehabilitation, the patient received a total of 117 visits of OT, 115 visits of PT, and 110 visits of SLP over 41 weeks of rehabilitation.

She displayed whole body ataxia as well as bilateral upper and lower extremity weakness with the left side weaker than the right. She required moderate assistance from her family for all ADL and transfers. The patient relied on a  percutaneous endoscopic gastrostomy (PEG) tube for nutrition due to risk of aspiration and received patching of the left eye to address her double vision and left eye palsy. It should be noted that in IPR the patient's right eye was patched as her right eye dysfunction was limiting her performance on functional retraining but in outpatient occupational therapy, retraining the visual musculature could be emphasized and the patch was switched to the left eye. Her cognitive status allowed her to follow multiple step commands to perform moderately complex tasks.

Occupational therapy evaluation, interventions and outcomes: Occupational therapy was initiated three times a week for 45 to 60 minutes per session. Her OT intervention initially focused on family education for home exercise program (HEP), core stability activities while laying supine on a mat with moderate to maximal assist, and unsupported sitting with moderate assistance. Improvement in ADL were monitored via the Patient-Specific Functional Scale (PSFS) and are noted in Table 2. During sitting she displayed frequent losses of balance and continued truncal ataxia. Scapular mobilization and kinesiology taping was utilized to encourage upright posture as well as to facilitate normalized motion of both arms. Monocular visual perception activities and tracking activities were utilized in conjunction with taping to the central portion of the patient’s left glasses lens to allow for continued peripheral vision and light input. 

**Table 2:** Outpatient Occupational Therapy Patient Specific Functional Scale PSFS scoring: 0 = inability to perform the activity at all; 10 = ability to perform the activity at the same level as before the injury or condition Key: w/c = wheelchair, UB = upper body, LB = lower body

	Outpatient evaluation	Month 1		Month 2	Month 3	Month 4	Month 5	Month 6	Month 7	Month 8	Month 9
Transfer from w/c to surfaces	1	2	4		5	6	6	7	6-7	8 (bed), 5 (toilet)	8 (bed), 7 (toilet)
Dressing UB/LB	1	3	4		5	6					
Toileting	0	3	4		5	6	6	6	7	7	7
Dressing UB							8	8	8	9	9
Dressing LB							5	6	8	8	8
Feeding self								6	8	8	9
Handwriting									2	4	4

By the second month, fine motor coordination (FMC) activities were initiated utilizing large sized pegs and beads requiring hand-over-hand assistance for fine motor tasks to facilitate normalized movement patterns secondary to ataxia (Table 3). Interventions also focused on modified ADL tasks with an emphasis on weight bearing through alternating upper extremities to incorporate into HEP. Neuromuscular re-education with weight bearing and proprioceptive neuromuscular facilitation (PNF) D1/D2 patterns were completed while sitting at the edge of the mat with minimal to moderate assistance to avoid loss of balance. During the third month, a resisted upper extremity exercise program was initiated with medium resistance elastic band with scapular retraction and shoulder horizontal abduction/adduction with trunk rotation to strengthen her core. She required minimal assistance to maintain her posture during these activities. She also participated in standing level neuromuscular re-education activities and gross motor coordination (GMC) tasks. The patient demonstrated an increased ability to self-correct her posterior loss of balance while in standing and this required minimal assistance while reaching minimally outside her base of support. A re-evaluation was completed after the third month which provided evidence of an increase in control and strength for completion of modified ADLs and transfers with minimal to moderate assistance with perturbations 50% of the time. 

**Table 3:** Outpatient Hand Function Results

Month of Testing (units in kg unless otherwise noted)	Hand grip testing (R/L)	Three Jaw Chuck (R/L)	Two point (R/L)	Lateral Pinch (R/L)	Nine Hole Peg (seconds) (R/L)	Box and Block Test (number of blocks) (R/L)
Outpatient evaluation	24/5	unable/ unable	unable/ unable	11/6	unable /195	N/A
1 month	24/8	8/5	7.5/4.75	11/7	280/141	N/A
3 months	36/25	11/8	9/6	14/11.5	150/48	12/19
6 months	N/A	N/A	N/A	N/A	111/56	14/22
9 months	44.3/35	15/11	12/8.5	18/12	60.6/39	17/26
10 months	53.3/ 36.6	15/11.5	12.5/8.5	18.5/13.5	85.7/38.3	17/24
Discharge - 10.5 months	54.3/33	17/13	13/10	20/14	87/39	16/24

During months four and five, she demonstrated an increase in core stability and improved coordination and control of her upper extremities. Core strengthening interventions included quadruped positioning on the mat with alternating arm-and-leg-lifts with minimal assistance as well as neuromuscular re-education techniques while seated on a foam pad reaching outside her base of support with minimal assistance to maintain balance. During FMC training with medium-sized pegs, she was overshooting 35% of the time. In addition, the patient performed light resistance clothespins and therapeutic putty to improve hand strength and dexterity.

By six months after initiation of outpatient OT, her upper extremity strength and core control became functional enough to focus on achievement of independence with basic self-care needs including dressing, toileting and feeding. Her interventions continued to include graded core and upper extremity strengthening and FMC/GMC tasks. The patient was introduced to modified techniques for dressing in supine as well as adaptive equipment for self-feeding as she was now able to eat with a modified diet and no longer required a feeding tube. Due to her continued need for a wheelchair for mobility, during months seven and eight, she received instruction in adaptation of kitchen and laundry tasks at the wheelchair level with emphasis on safety and adapted performance. She also started a writing program with her non-dominant left hand as coordination was improving faster than her right hand. Through months nine and ten, interventions continued to focus on modified techniques and safety with both basic and advanced ADLs as well as functional transfers and independence at wheelchair level in preparation for discharge.

Physical therapy evaluation, interventions and outcomes: Physical therapy was initiated in conjunction with OT and SLP, three times a week for 45 minute sessions each. Upon initial evaluation, the patient’s Postural Assessment for Stroke Scale (PASS) score was 11/36 (0= maximal disability, 36= no disability). Interventions for physical therapy focused on dynamic and static sitting and standing balance activities, pre-gait and family education. Improvement in physical mobility were monitored via the Patient-Specific Functional Scale (PSFS) and are noted in Table 4.

**Table 4:** Outpatient Physical Therapy Patient Specific Functional Scale (PSFS) PSFS scoring: 0 = inability to perform the activity at all; 10 = ability to perform the activity at the same level as before the injury or condition Key: w/c = wheelchair, ADL = activities of daily living, min = minimal assistance, SBA = standby assistance

Activity	Month 6	Month 7	Month 8	Month 9
Independent bed mobility: supine to sit	9	10	10	10
Gait with U-step with assist	7	7	8	9
Gait with U-step up and down 6-8 inch step in home	0	1	3	5
Standing balance unsupported with assist	3-4	4	5	7
Transfers: w/c to mat	7-8	8-10	9-10	9
Car transfer	9	10	8	10
Ascend stairs with bilateral handhold	7 (min)	5 (SBA)	6 (SBA)	6 (SBA)
Down stairs with bilateral handhold/sideways	6 (min)	3-4 (SBA)	5 (SBA)	6 (SBA)
Sit to stand from w/c using U-step			3 (SBA)	5 (SBA)
Standing dynamic balance for ADL			1 (SBA)	1

Therapeutic activities included unsupported trunk rotation with weighted ball and seated marching with alternating upper extremity requiring moderate assistance for support and upright posture. The patient also participated in parallel bar side-stepping with moderate assistance at the hips to decrease overstepping. A rolling walker weighted with 2.27 kg (5 lbs) was utilized during gait training with moderate assistance for 46 m (150 ft). She also demonstrated standing pivot transfers with moderate assistance with external perturbations. After one month, her PASS score was 26/36. Months two and three focused on core stability exercises in supine and quadruped via crawling forward and backward, supine-to-kneeling and mini push-ups with moderate assistance. Interventions also focused on fall recovery as the patient demonstrated a risk of anterior loss-of-balance from the toilet at home. Her timed-up-and-go score progression is noted in Table 5.

**Table 5:** Timed Up and Go (TUG) Results

Month	Time and relevant details
2 months	131 seconds with rolling walker and partial/moderate assist
3 months	100 seconds with rolling walker and supervision/touching assist
5 months	71 seconds with rolling walker and supervision/touching assist
6 months	91 seconds with U-step walker and partial/moderate assist
6.5 months	72 seconds with U-step walker and partial/moderate assist
7 months	78 seconds with U-step walker and supervision/touching assist (increased time, however improved motor control and less physical assistance needed with turns)
8 months	63 seconds with U-step and supervision/touching assist
9 months	59 seconds without assistive device and supervision/touching assist which increased to parttial/moderate assist during turning
10 months	51 seconds with no assistive device and supervision/touching assist

At four months, her PASS score was 28/36 and was not repeated after this date. Pre-gait in the parallel bars was advanced to incorporate single leg stance, step tap with alternating lower extremities in both the ascending and descending direction; her anterior step-downs and posterior step-ups with external perturbations required moderate to maximal assistance for fall prevention. As her core stability increased in months five and six and she was no longer falling forward during transitional movements, her gait training progressed to outdoor, uneven surfaces, and stair training. At the beginning of month five, the patient’s Berg balance score was 13/56 (56 = normal balance) and by the end of the sixth month, it had improved to 17/56 and was no longer reported in the medical record. More advanced strengthening and balance activities were initiated including lunges, squats, standing ball bouncing and tandem weight shift with minimal assistance to maintain balance. She was also started on a program with the LiteGait and treadmill as well as an elliptical machine. By month seven, the patient was started on a U-step walker (In-Step Mobility Products, Skokie, IL, 60076. www.ustep.com) to aid in ambulatory control and balance. Transfer training shifted focus toward family education and safety to prevent falls. Months eight through ten focused on core strength and stability, lower extremity strengthening, gait training with the U-step with minimal assistance from family, and independence at wheelchair level with self-propulsion. 

**Figure 9:**
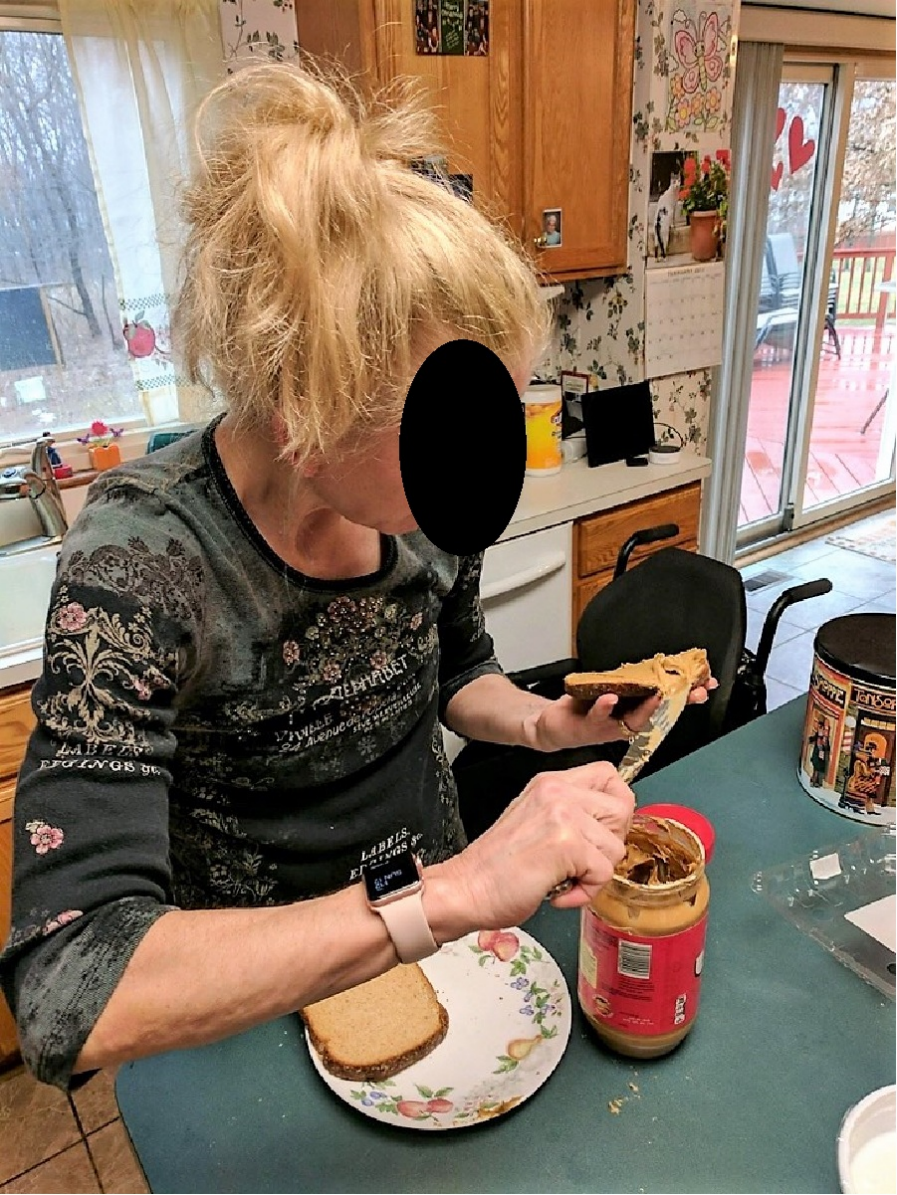
Standing-Level Meal Preparation Patient demonstrating ability to complete independent meal preparation in her own home

By month ten, the patient had started to plateau with her functional level and was being prepared for discharge. Overall she progressed in her independence for both basic and advanced ADL and transfers. She was able to self-propel her wheelchair independently. Initially requiring moderate to maximal assistance, her transfers to and from common household surfaces (bed, toilet and kitchen chair) now only required steadying assist/close supervision. She demonstrated increased core stability and upper extremity control and coordination to participate with upper and lower body dressing and toileting needs which only required steadying assist (she initially required moderate to maximal assist). She was able to statically stand while leaning her trunk against a counter to participate with light advanced ADL tasks such as folding laundry and simple meal preparation with close supervision (Figure 9). She was able to propel her wheelchair around the house and assist with setting and clearing the table for meals. She resumed modified leisure activities such as working in her garden, painting and bike riding. She was able to utilize the U-step walker with minimal assistance from her family and caregivers to move around the house and community with good tolerance for activity. She continued to require support and supervision at home from family and hired assistance to ensure safety although she was able to function in her environment at the wheelchair level with little to no assistance. 

## Discussion

The purpose of this case study was to describe the longitudinal management and outcomes for OT and PT for a patient with a cerebellar stroke with surgical excision over a 14-month treatment course. This patient had an exceptionally long and complex continuum of care that required close coordination and facilitation between disciplines and care settings. A goal of this case report was to provide rehabilitation clinicians with a long-term perspective and understanding of the course of recovery for a patient after cerebellar cerebrovascular accident or related injury. Kelly et al. [4] examined outcomes after cerebellar stroke in 58 patients. The results of this case study are consistent with this finding as this patient demonstrated significant recovery, however, it did not appear that the patients in the Kelly study had such an initially complex ICU and surgical course.

The patient’s early recovery and ability to begin early mobility in the ICU may have been hastened by the implementation of MSS. In the acute care setting, MSS can promote brain reorganization specifically targeting the reticular activating system (RAS) [7]. The RAS is critical due its’ role in consciousness, attention, sleep/wake cycle, motivation, and physiological function. These interventions are aimed at facilitating the patient through the levels of recovery with the intention of improving prognosis and earlier rehabilitation [8]. The populations that benefit from multimodal sensory stimulation include patients in the ICU who are receiving minimal to no sedative medications and are physiologically responsive. Appropriate diagnoses to employ MSS include patients with a neurological injury that result in a change of baseline of the patient such as a stroke, traumatic brain injury, anoxic brain injury, and encephalopathy. Prior to administering MSS, it is important to determine that the client is medically stable enough to tolerate the services and that the environment (noise, lighting, temperature) is able to be controlled [5]. Treatment includes stimulating the auditory, visual, tactile, olfactory, gustatory and kinesthetic systems. Components of MSS may include speaking, ringing a bell, exposing the patient to the smell or taste of fruit, light moving touch on varying parts of the body and purposeful movement in the early stages of medical stability after the neurological insult. It is important to have an occupational profile to ensure that treatment includes stimulation that is meaningful to the client.

Within the rehabilitation literature, it is well established that early mobility in the ICU is effective, shortens length of stay and provides better and more cost effective outcomes for the ICU [6]. Early mobility in the ICU is often predicated on the patient being able to be physically, psychologically and medically stable enough to participate [7]. It should be noted that there is a dearth of literature on early mobility regarding brain injury rehabilitation, especially cerebellar stroke. The early initiation of MSS was felt to be a key component in assisting in the early restoration of purposeful cognitive status to be able to follow instructions for early mobility in the ICU. For these services, the physical therapist had daily communication and rounded with the occupational therapist who participated in the MSS. Once appropriate medical and cognitive stability was achieved through MSS then physical therapy was initiated. This close coordination of care may be an effective management strategy of the use of physical therapist and occupational therapist skill sets especially in the era of limited personnel resources within the hospital.

A key component of this patient’s positive outcomes after such a long and intensive rehabilitation process was likely the strong social and economic support network available to her. Throughout the entire rehabilitation process, the patient’s husband and children were actively involved and frequently assisted in and participated with treatments in all rehabilitation settings. Even after discharge from outpatient PT/OT, the patient’s spouse set up an unweighting harness for her home treadmill by installing asupport system in the ceiling of their living room. The patient also had adequate and appropriate insurance to cover extensive rehabilitation services especially for such a long duration of time. Another factor in the patient’s recovery was the absence of serious medical complications post-surgically, allowing for consistent, progressive, aggressive rehabilitation. Finally, the patient was a high-level athlete prior to her injury and the patient translated this work ethic and response to adversity in her recovery. This may have provided her with the intrinsic motivation to continue to participate in rehabilitation when other patients may have not applied that intrinsic motivation and achieved as significant of outcomes.

Unilateral eye patching during acute neurological rehabilitation remains controversial with limited evidence of clinical efficacy with some investigators noting that acute eye patching may limit long term vision recovery [9]. Although there is no consensus on eye patching, patient care in the IPR environment significantly emphasized an expedited return to safe function at home. During early attempts to rehabilitate the patient’s diplopia, her vision was not demonstrating significant recovery and was delaying rehabilitation of functional mobility and ADL retraining. After this finding, the physiatrist, care team, patient, and family determined that the patient’s diplopia was a primary barrier to restoration of safe ADL. This was anticipated to increase the IPR length-of-stay and delay safe discharge home to outpatient OT and formal vision rehabilitation [10]. The patient was educated in the relative risks and benefits of patching vs. not patching and she preferred to focus on functional recovery utilizing the eyepatch. After discharge from IPR, outpatient OT was initiated which included a vision rehabilitation component and employing a transparent central field patch to facilitate scanning and ambient light. This area of practice would benefit from further research into acute eyepatching and its impact on functional recovery in the presence of severe disability.

Limitations and future research

As this case report focused on one highly motivated and healthy patient along the entire continuum of care, outcomes may not be generalizable to other patients with similar diagnoses. In addition, although standardized, reliable functional outcome measures were employed across the continuum of care, there was not consistency of use of a single functional outcome to track and trend the patient’s functional recovery over her entire rehabilitation journey as each therapist applied what he or she anticipated to be the most clinically applicable and sensitive tool at that point in the patient’s recovery. Future research about this diagnosis should examine outcomes after standard care without strong interdisciplinary communication and collaboration as compared to highly coordinated, facilitated interdisciplinary care and between care settings. Finally, further examination of the applicability and validity of commonly utilized stroke outcome measures (PASS, National Institute of Health Stroke Scale, etc.) should be evaluated for patients with cerebellar involvement.

## Conclusions

This patient demonstrated substantial gains and recovery after a life threatening cerebellar stroke with an initially poor survival prognosis. In the early stages of rehabilitation, multimodal sensory stimulation closely coordinated with early mobility in the ICU were utilized to assist with eliciting a state of arousal from a comatose state to transition to the inpatient rehabilitation unit. Although the primary mean of mobility was via wheelchair, the patient was able to return home and be independent in modified ADL and instrumental ADL.
